# Mobile EEG in research on neurodevelopmental disorders: Opportunities and challenges

**DOI:** 10.1016/j.dcn.2019.100635

**Published:** 2019-03-08

**Authors:** Alex Lau-Zhu, Michael P.H. Lau, Gráinne McLoughlin

**Affiliations:** Social, Genetic and Developmental Psychiatry Centre, Institute of Psychiatry, Psychology and Neuroscience, King’s College London, London, United Kingdom

**Keywords:** EEG, ERP, Biomarker, Neurodevelopmental disorders, Developmental psychopathology

## Abstract

Mobile electroencephalography (mobile EEG) represents a next-generation neuroscientific technology – to study real-time brain activity – that is relatively inexpensive, non-invasive and portable. Mobile EEG leverages state-of-the-art hardware alongside established advantages of traditional EEG and recent advances in signal processing. In this review, we propose that mobile EEG could open unprecedented possibilities for studying neurodevelopmental disorders. We first present a brief overview of recent developments in mobile EEG technologies, emphasising the proliferation of studies in several neuroscientific domains. As these developments have yet to be exploited by neurodevelopmentalists, we then identify three research opportunities: 1) increase in the ease and flexibility of brain data acquisition in neurodevelopmental populations; 2) integration into powerful developmentally-informative research designs; 3) development of innovative non-stationary EEG-based paradigms. Critically, we address key challenges that should be considered to fully realise the potential of mobile EEG for neurodevelopmental research and for understanding developmental psychopathology more broadly, and suggest future research directions.

## 1 Introduction

Neurodevelopmental disorders, such as attention-deficit/hyperactivity disorder (ADHD) and autism spectrum disorder (ASD), are known to impact brain function across the lifespan (Boivin et al., 2015; Thapar et al., 2017), driven by a complex interplay between genetic and environmental influences. Efforts have been dedicated to the search for biological markers (or biomarkers) of these conditions (Insel et al., 2010), including those that may be along the pathway from genetic/environmental influences to behavioural symptoms (Meyer-Lindenberg and Weinberger, 2006; Thapar et al., 2017; Walters and Owen, 2007). Sensitive and specific brain-based markers of atypical development are likely to inform optimised interventions (e.g., earlier or more targeted), potentially mitigating life-long difficulties typically associated with neurodevelopmental conditions.

The last decade has observed a proliferation of mobile sensing technologies (Trull and Ebner-Priemer, 2013; Wilhelm and Grossman, 2010). Among these, emerging state-of-the-art electroencephalography (EEG) tools now enable flexible recording of brain activity in real-time. We refer to these collectively as *mobile EEG*. These advances have initially been driven by an interest in incorporating real-time neural recording into consumer-oriented applications, including the development of brain-computer interfaces (or BCI) for a vast range of applications such as gaming control (Liao et al., 2012a) and drowsiness detection during driving (Lim et al., 2014). Other areas of interest include marketing (Lee et al., 2007), architectural and urban design (Karandinou and Turner, 2017), and personalised health, for instance, sleep monitoring (Quante et al., 2018) and ‘brain-training’ (Maskeliunas et al., 2016; Wei et al., 2017). These developments have fuelled the need for forms of EEG that are increasingly more available and appealing to everyday users, ideally without specialist researchers. Consequently, the field of EEG has observed dramatic advances in the last decade which have further promoted its scientific utility. Interest in this technology has already prompted special issues dedicated to its neuroscientific use within several prominent academic journals (De Vos and Debener, 2014; Gramann et al., 2014b), yet its relevance for neurodevelopmentalists remains little discussed.

In this review, we highlight the opportunities afforded by mobile EEG to overcome limitations of traditional neuroimaging modalities for studying neurodevelopmental disorders. If optimally implemented, the incorporation of mobile EEG promises to illuminate developmental psychopathology mechanisms and facilitate the identification of putative brain-based biomarkers. Although promising, the use of mobile EEG in neurodevelopmental research is still in its infancy, thus we also discuss key challenges lying ahead.

## 2 Recent advances

Human EEG was first recorded by Hans Berger in 1924, representing one of the oldest non-invasive tools to record brain activity in real-time. EEG primarily captures summed electrical field activity (measured in voltage) produced by pyramidal cortical neurons that are aligned parallel to the scalp.

EEG has excellent temporal resolution, but relatively poorer spatial resolution (Table 1). Traditional EEG systems have a long-standing history in neurodevelopmental research (Loo et al., 2015; McLoughlin et al., 2014a) but are typically restricted to the laboratory (e.g., involving heavy amplifiers and extensive wiring), thus potentially limiting the populations that can be readily studied and the research questions that can be addressed, as we expand on later (see Section 3).

**Table 1:** Comparison between Mobile EEG and Other Functional Brain-Imaging Techniques.

	fMRI	PET	MEG	Traditional EEG	Mobile EEG	Additional Notes on Mobile EEG
Temporal resolution	1 sec	30-40 sec	1-4 msec	1-4 msec	1-4 msec	Issues with delay/jitter need to be corrected (e.g., by the manufacturers or with additional offline alignment); see Section 4.1.2
Spatial resolution (mm)	1- 5	2	1-5	20-30 (scalp) to 5-10 (source)^a^	Comparable to traditional EEG	Low-density systems provide less spatial information/limit source-based analyses compared to high-density systems; see Section 4.2
Neural signal	Indirect	Indirect	Direct	Direct	Direct	Effective artefact rejection is critical (particularly non-stereotyped motion-based artefacts); see Section 4.5
Invasiveness	No	Yes	No	No	No	The study of certain time-sensitive cognitive processes might become more feasible (e.g., in the immediate aftermath of traumas/stressors); see Section 3.1
Set-up time (min)	30-60	60	30-60	30-60	5-20	Some of the systems could reduce set-up time (e.g., dry electrodes) but might be less comfortable for participants; see Section 4.3
Estimated cost per participant ($)	930	1900	820	45	20-45	Consumer-grade/low-density systems can be inexpensive (e.g., <$1k), whereas research-grade/higher-density systems currently can be similar in price to setting up a whole traditional EEG lab (e.g., >$20k); see Table 2
Testing setting	Research/clinical	Primarily clinical	Research/clinical	Research/clinical	Flexible (e.g., home visits)	Standardization protocols are advised for large-scale testing in everyday settings; see Table 3
Task paradigms	Stationary	Stationary	Stationary	Stationary	Stationary; motion-based; in everyday settings	Motion-based paradigms are currently focused on neurotypical populations; see Section 3.3

*Note*. FMRI = functional magnetic resonance imaging; PET = positron emission tomography; MEG = magnetoencephalography; EEG = electroencephalography.

a Typically needing high-density montages with subject-specific forward model (Akalin Acar and Makeig, 2013).

Several terms associated with mobile EEG have been used in this rapidly expanding literature (e.g., portable/wireless/wearable/dry EEG). As this field consolidates, terminology used by neurodevelopmentalists is likely to become more consistent (Bateson et al., 2017). For example, the concept of ‘transparent EEG’ has recently been introduced to describe the combination of features deemed necessary for everyday mobile sensing applications, such as the system also needing to be self-applicable, motion-tolerant, near invisible and suitable for long recordings (Bleichner and Debener, 2017). Here we use the umbrella term mobile EEG to highlight the two key novel aspects of mobility (of the systems and of the participants) and their relevance for neurodevelopmental research.

### 2.1 Hardware

**Fig. 1:**
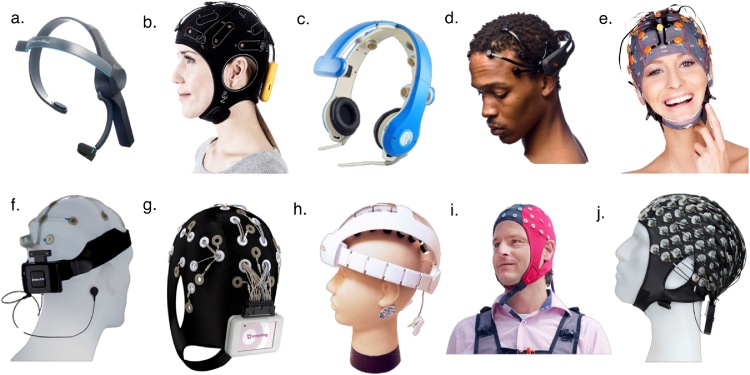
Examples of some mobile EEG systems. See Table 2 for an overview of key technical specifications.

Mobile systems encompass hardware solutions that are transforming EEG into one of the most accessible neuroscientific tools (Fig. 1 and Table 2). These systems often consist of small, light-weight amplifiers alongside wireless transmission, which contribute to the devices’ increased portability. These devices can be ‘wearable’. For example, participants could in principle stand up and walk freely at any point, with some systems allowing for relatively long recordings or even self-fitting. However, given the diversity of manufacturers – for both research-grade systems (tailored to scientific research) and consumer-oriented systems (targeted primarily for everyday applications) – not all of these features are necessarily present in a given system. Thus, the degree of the systems’ mobility can vary, such that some devices need to be carried within a backpack while others are fully head-mounted (Bateson et al., 2017).

**Table 2:** Overview of Key Technical Specifications for Example Mobile EEG Systems.

System (manufacturer)	Density (channels)	Electrode type	Resolution (bits)	Maximum sampling rate (Hz)	Bandwidth (Hz)	Weight (g)	Battery life (h)	Wireless transmission	Supported platforms^a^
a. MindWave (NeuroSky)	1	Dry (stainless steel)	12	512	1–100	90	6-8	Bluetooth	Windows, Linux
b. ENOBIO 8 (Neuroelectrics)	8	Wet (gel)	24	500	0-125	65	6-23	Bluetooth/Wifi	Windows, Mac OS
c. BR8+ (BRI)	8	Dry (spring, foam)	24	500	0.12-125	269	10	Bluetooth	Windows
d. EPOC (Emotiv)	14	Wet (saline)	14-16	128/256	0.16-43	116	6-12	Bluetooth	Windows, Mac OS, iOS, Android
e. g.SAHARA (G.Tec)	16	Dry (pins, metal)	24	500	0-40	233	10	Wifi	Windows
f. B-Alert X24 (Advanced Brain Monitoring)	20 (+4 auxiliary)	Wet (gel)	16	256	0.1-100	110	8-15	Bluetooth	Windows
g. Smarting (mBrainTrain)	24	Wet (gel)	24	250/550	0-250	60	5	Bluetooth	Windows, Linux, Android
h. Trilobite (Mindo)	32	Dry (spring, foam)	24	500	0.23–1300	524	10	Bluetooth	PC, Android
i. eego sports (ANT Neuro)	64	Wet (gel)/ Dry (polymer)	24	2048	0–532	500	5	Wired to a tablet (in a backpack)	Windows, Linux
j. Mobile-128 (Cognionics)	64/128 (+8 auxiliary)	Wet (gel)	24	500	0–131/262	460	6–8	Bluetooth	Windows, Linux, Mac OS

*Note.* These systems are depicted in Fig. 1.

a These would determine the additional hardware requirements and hence the overall weight of the recording kit; labs with the necessary engineering expertise may adapt systems to build custom applications on new platforms.

Recent developments in dry electrodes (Liao et al., 2012b) could further decrease preparation time, removing the need to apply conductive gel/saline patch and prepare the skin as required in traditional EEG to reduce skin-electrode contact impedance (though not without limitations; see Section 4). Some dry electrodes involve ring-like structures with pins to reach the scalp through the hair (Hairston et al., 2014), although others use foam-based materials wrapped in conductive fabric materials (Lin et al., 2011). An exciting possibility includes non-contact electrodes, which involve amplifying the weak bio-potential without requiring electrode-skin contact at all. These have been applied recently for detecting foetal electrocardiogram signals in pregnant women, using only a small non-contact sensor, non-invasively and in home settings (Ho et al., 2017; Sharma et al., 2017). Similar non-contact technologies are also being developed for EEG (e.g., Chi and Cauwenberghs, 2010).

Complementary to advances in EEG recording, more convenient means for task stimuli presentation are now available for everyday settings, using lightweight and small Raspberry Pi 2 computers (Kuziek et al., 2017), smartphones (Debener et al., 2015; Stopczynski et al., 2014b), tablets (Griffiths et al., 2016), and augmented-reality eyewear (Duvinage et al., 2013). These mobile presentation-devices can be coupled with tailored commercially-available software programmes that allow for data collection with high temporal precision (e.g., Presentation Mobile App; www.neurobs.com). Some of the smartphone-based applications can incorporate stimuli presentation, signal recording and online processing all within a single device (Blum et al., 2017; Stopczynski et al., 2014b; Wang et al., 2013).

### 2.2 Signal processing

The past decade has observed significant advances in analytical approaches to EEG data, making EEG even more attractive scientifically and increasing the need for more practical systems. EEG data have been traditionally analysed in the frequency domain (i.e., extracting frequency bands typically between 1–70 Hz) and time domain (i.e., identifying time- and phase-locked EEG activity to a stimulus, i.e., event-related potentials or ERPs); more recent techniques combine both approaches, including time-frequency analyses (Buzsáki, 2006; Makeig et al., 2004).

Powerful computational tools – many of which can be exploited in mobile EEG studies – are beginning to improve EEG’s ability to reveal underlying brain source dynamics. For example, a spatial filtering technique known as independent component analysis (ICA) can be used to separate temporally and functionally independent components into brain and non-brain sources (Delorme et al., 2012; Delorme and Makeig, 2004). ICA’s utility for analysing mobile EEG data has already been demonstrated for both artefact correction (including ocular artefacts) and source-based analyses (Debener et al., 2015; Ehinger et al., 2014; Gramann et al., 2014a; Kontson et al., 2015). Moreover, advances in source separation have improved the localisation accuracy of EEG sources, though typically necessitating high-density montages and subject-specific anatomically-precise forward models (Akalin Acar and Makeig, 2013), hence high-density mobile EEG systems would be needed to take advantage of these advances (see later Section 4.2). ICA has shown to be informative for neurodevelopmental research using traditional EEG systems (Lenartowicz et al., 2014; McLoughlin et al., 2014b; Milne et al., 2009) and for big-data automated EEG pre-processing pipelines (Bigdely-Shamlo et al., 2015; Chaumon et al., 2015; Mognon et al., 2011). Such methods could also be harnessed for large-scale EEG-based neurodevelopmental studies now facilitated by mobile applications (see Section 3.2).

Novel analytical methods are increasingly being exploited to gauge functional brain networks (and their interactions) using EEG data; for a comprehensive review readers can refer to existing coverages (Bridwell et al., 2018; Sakkalis, 2011; Stam, 2005). One example is the use of connectivity measures that are based on synchronisation of EEG activity (Sakkalis, 2011), with applications in autism (O’Reilly et al., 2017; Tye and Bolton, 2013) and schizophrenia (Maran et al., 2016). Nonlinear signal processing methods are also being increasingly applied (e.g., single-trial transient events and non-sinusoidal fluctuations) (van Ede et al., 2018). Such approaches, compared to traditional spectral analyses, promise to extract more functional information (with greater sensitivity) to unveil biomarkers in neurodevelopmental disorders (Bosl et al., 2018; Tierney et al., 2012).

## 3 Key opportunities

Although mobile EEG research is in its infancy, this technology has already been used by cognitivists to study a variety of processes such as attention (Jungnickel and Gramann, 2016), memory (Griffiths et al., 2016; Piñeyro Salvidegoitia et al., 2019), spatial cognition (Ehinger et al., 2014), speech/auditory processing (Callan et al., 2015; Mirkovic et al., 2016) and motor processing (Lin et al., 2016; Lo et al., 2016; Wong et al., 2014). Mobile EEG has also been used for everyday applications in sports (Park et al., 2015), urban behaviours (Mavros et al., 2016), emotion recognition (Aspinall et al., 2015; Bercik et al., 2016; Li et al., 2015), neurofeedback (Stopczynski et al., 2014b), motor rehabilitation (Kranczioch et al., 2014; Wagner et al., 2012), epilepsy (Askamp and van Putten, 2014), and cognitive impairment (Kashefpoor et al., 2016). The explosion of studies employing mobile EEG, although predominantly in neurotypical populations, signals similar opportunities for neurodevelopmental research.

### 3.1 Increased ease of use in neurodevelopmental populations

EEG has a long tradition of superior practicality and flexibility as a functional brain method in children and individuals across ages and abilities (Loo et al., 2015; McLoughlin et al., 2014a), and the same appears to be the case for mobile EEG as exemplified by a recent case study investigating auditory brain responses in a minimally-verbal child with cerebral palsy (Yau et al., 2015). Mobile EEG could even better cater for the cognitive, sensory, and/or motor sensitivities that characterise various neurodevelopmental conditions. For instance, participants with ADHD may struggle to stay still for an extended period, and participants with a learning disability could find long assessments demanding. Mobile EEG offers a solution to these issues with the possibility of high-quality EEG data via maximally flexible testing protocols. Some mobile systems require as little as 5–10 minutes preparation which provides the possibility of more breaks and allows for more interruptions with minimal impact on the data. Miniature and concealed forms of EEG could minimise drop-out of those who find electrode-scalp contact uncomfortable for an extended period (e.g., autistic participants with sensory hypersensitivities) along with an improvement in aesthetics (Debener et al., 2015).

Mobile EEG could increase the feasibility of neonate neuroscientific research. A proof-of-concept study successfully applied an 8-electrode mobile EEG system in a clinical setting to monitor sleep states in six nonclinical neonate, and also seizure-related activities in two neonates with congenital abnormal cortical development (Demene et al., 2017). Mobile EEG may also facilitate combination with complementary modalities, such as functional near-infrared spectroscopy (fNIRS) (Safaie et al., 2013), which measures hemodynamic responses and is increasingly popular in cognitive/developmental research as this technology is also portable/wearable/wireless (Mazzoni et al., 2018; Pinti et al., 2018).

The flexibility of mobile EEG has been demonstrated in several existing studies (Liu et al., 2013; Poulsen et al., 2017; Wascher et al., 2014), including one in an open cockpit biplane during flight (Callan et al., 2015). While most commercially-available mobile EEG systems cannot be operated with smartphones – with a few exceptions (Blum et al., 2017; Stopczynski et al., 2014b; Wang et al., 2013) – smartphone-based EEG holds promise for further increasing the portability of a ‘mobile EEG lab’ that would allow for controlled stimuli-delivery in everyday settings with minimal equipment. Testing in convenient locations (e.g., schools or homes) can be advantageous for neurodevelopmentalists, as this reduces the burden on participants and their families from travelling, and is also more inclusive of those who would prefer not to travel, for example, autistic participants who may be anxious about travelling or patients for whom leaving a hospital could be counterindicated. For certain time-sensitive neurocognitive processes, mobile EEG might be preferred. For instance, trauma victims may find noisy scanners/confined spaces/extensive wiring (inherent to traditional neuroimaging) undesirable, particularly soon after trauma (e.g., up to 6 hours post-trauma) – a putative critical time window for trauma memory consolidation (McGaugh, 2015). Mobile EEG could lead to new possibilities to study this process in real-life traumas/stressors, in contrast to prevailing studies using laboratory ‘trauma’ instead (James et al., 2016; Lau-Zhu et al., 2018).

### 3.2 Integration into developmentally-informative research designs

Mobile EEG could facilitate large-scale studies (though not without challenges; see Section 4.4). Powerful designs for neurodevelopmental research incorporate longitudinal and/or genetically-informative elements (e.g., twin/family studies) alongside cross-disorder comparisons (Kendler and Neale, 2010; Lau-Zhu et al., 2019), to illuminate causal directions and identify converging/diverging neurodevelopmental pathways. One study used a commercial EEG system to acquire data from 400 people under only three months in a museum setting (Kontson et al., 2015), demonstrating the potential increased in efficiency of EEG-based data collection. Another study involved four daily mobile EEG recordings in the home environment (Zich et al., 2015) or even every other day over a month (Zich et al., 2017) to train motor imagery, illustrating again the feasibility of highly-frequent mobile EEG-based assessments (e.g., within longitudinal designs), which could be facilitated with future self-fitting systems.

Mobile EEG could be integrated seamlessly into research centres (de Wit et al., 2017), stimulating interdisciplinary collaborations in psychopathology research, such as combining neuroscience with epidemiology/genetics (McGuffin and Plomin, 2004), as well as data collection in non-western samples worldwide to broaden the study of sociocultural factors in developmental neuroscience (Choudhury, 2010). Additionally, mobile EEG could promote cross-site collaborations, joining efforts for large-sample studies with independent replications that can be transformative for the field, in light of recent controversies with replicability in neuroscience (Carp, 2012). Robust mobile EEG data, alongside source-resolved EEG analytical approaches (Makeig et al., 2004), could serve as a foundation for targeted analyses with subgroups using more expensive, invasive and/or multimodal imaging (Bridwell et al., 2013; McLoughlin et al., 2014a). Furthermore, sensitive EEG-based genetic risk markers derived from well-powered studies may inform greater phenotypic specificity for more targeted molecular genetic studies (McLoughlin et al., 2018), and uncover gene-behaviour neurodevelopmental pathways (Anokhin, 2014; Walters and Owen, 2007).

### 3.3 Development of innovative neurocognitive paradigms

**Fig. 2:**
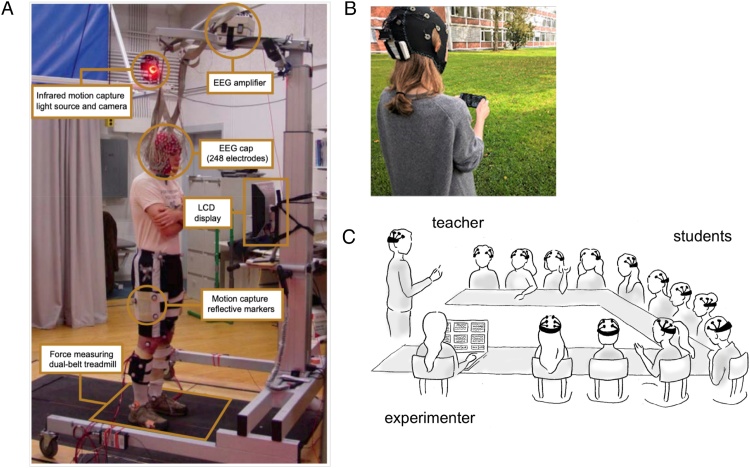
Illustration of innovative EEG-based paradigms facilitated by ongoing mobile EEG developments. Examples include a set-up a) for mobile brain/body imaging (MoBI) allowing for body movements merging EEG with other motion-based sensors, adapted from Gramann et al. (2010); b) using mobile EEG while walking with smartphone-based stimuli presentation (of word stimuli) in an outdoor setting (i.e., a pre-specified route), adapted from Piñeyro Salvidegoitia et al. (2019); c) using mobile EEG simultaneously in multiple individuals within an indoor social setting (i.e., in the classroom), adapted from Dikker et al. (2017).

An exciting and unprecedented opportunity is the assessment of brain activity during novel paradigms where participants’ movements are allowed (Fig. 2). For example, some recent studies with typical populations have used mobile EEG during lab-based simulations of real-life events, such as in a driving simulator (Gevins et al., 2012), flying simulator (Callan et al., 2015), shop-browsing simulation (Bercik et al., 2016), as well as simulation of a social gathering (Gevins et al., 2012; Poulsen et al., 2017). There is enormous potential for novel paradigms to improve understanding of atypical cognitive and affective processes. For instance, while several neurodevelopmental conditions are associated with difficulties in social interactions and communication, paradigms that aim to probe for these difficulties tend not to involve an interaction element. Creative approaches have begun to incorporate EEG in naturalistic interactions in dyads (Kinreich et al., 2017; Leong et al., 2017; Liao et al., 2015) or even multiple individuals simultaneously known as ‘hyperscanning’ (Babiloni and Astolfi, 2014; Dikker et al., 2017). Mobile EEG systems might facilitate adaptation of such paradigms into neurodevelopmental populations and everyday settings.

An approach coined as mobile brain/body imaging (MoBI) has pioneered the development of EEG-based ecologically-valid paradigms incorporating participants’ physical actions (e.g., walking or pointing) (Beurskens et al., 2016; De Sanctis et al., 2014; Gramann et al., 2014a; Makeig et al., 2009; Wagner et al., 2016). To this end, mobile EEG has been combined with bicycles (Zink et al., 2016), walkways (Beurskens et al., 2016), body-sensing technologies (Cruz-Garza et al., 2014; De Sanctis et al., 2014) and virtual reality (Banaei et al., 2017; Ehinger et al., 2014), resulting in relatively complex laboratory set-ups to model real-world scenarios. Similar approaches could inform naturalistic paradigms for neurodevelopmental research where atypical movement-related processes are implicated, including gesture development (Capone and McGregor, 2004) and developmental coordination disorder (Visser, 2003). While promising, developing such paradigms for neurodevelopmental research is likely to bring additional challenges too (Section 4.5).

## 4 Current challenges

While the application of several mobile EEG advances in neurodevelopmental research is now feasible, a number of challenges lie ahead for their maximal exploitation. We refrain from evaluating specific systems, given the ever-growing number of manufacturers and rapid developments in this area – evaluation of a specific system now may not be relevant in the near future (e.g., a given headset with its specifications may no longer be produced). Ultimately, any system could be useful depending on specific circumstances, including the research question, intended analyses, targeted population, experimental paradigm, expertise involved and relationships to the manufacturers. Hence, we raise general issues – including those that could inform industry (e.g., in designing specific headsets tailored to neurodevelopmental research) – and suggest future directions to increase the utility of this new technology for developmental psychopathology research.

### 4.1 Signal quality

Of utmost importance is the signal quality afforded by these new systems, to decide on their scientific appropriateness and better evaluate trade-offs with costs. Despite the proliferation of this technology, often little information about signal quality is provided. Nevertheless, we summarise some emerging findings regarding validity and reliability of mobile EEG data.

#### 4.1.1 Validity

**Fig. 3:**
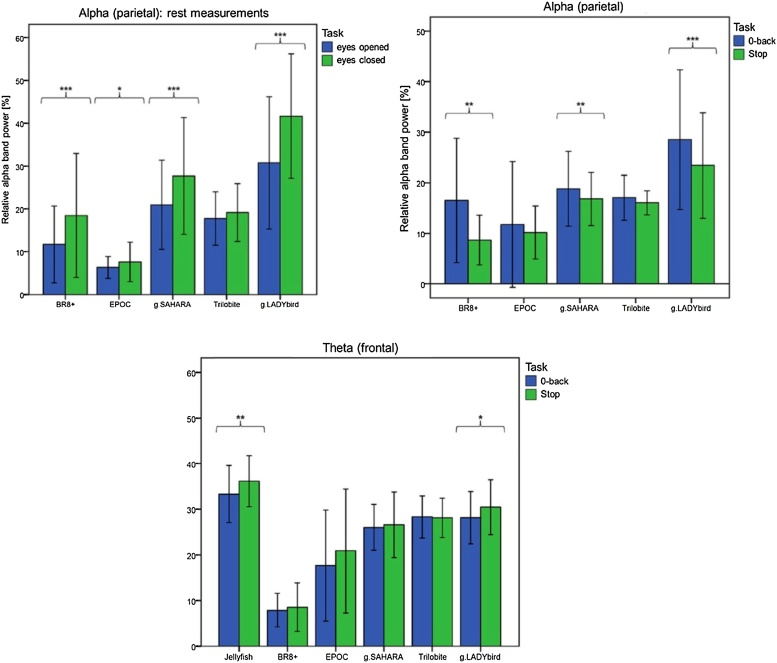
Example of spectral features detected by different mobile EEG systems (***: *p* ≤  .001; **: .001 < *p* ≤  0.01; *: .01 < *p* ≤  .05; error bars indicate ±1 *SD*). Top panels show that, as expected, parietal alpha-band power significantly increased from an eyes-opened to an eyes-closed resting condition (except for *Trilobite* – a dry system), and from an easy to a more demanding cognitive-task condition (except for *Trilobite* and *EPOC*, the latter being a saline-based system). Bottom panel shows that the expected frontal theta-band power significantly increased from an easy to a more demanding cognitive-task condition only in two systems, one which is research-grade and gel-based (*g.LADYbird*), and one dry system (which only had frontal electrodes called *Jellyfish*). Results were aggregated across a selection of electrodes depending on the specific configuration of each system. Depiction of some of these systems can be found in Fig. 1. Adapted from Radüntz (2018).

Most available studies use traditional EEG systems as the ‘gold-standard’ for reference. One type of study shows that mobile systems are able to replicate findings derived from traditional EEG (providing support for construct validity), such as capturing several expected ERPs (N2, P3 and reward-positivity) during well-established lab-based stationary cognitive paradigms (Krigolson et al., 2017). Expected frequency-based features can also be extracted, for example, reduction in theta power and increase in alpha power from eyes-closed to eyes-open resting conditions (Debener et al., 2015), even in a single-channel dry system (Johnstone et al., 2012), as well as event-related decrease in beta power for successful memory retrieval in a outdoor walking-based paradigm (Griffiths et al., 2016; Piñeyro Salvidegoitia et al., 2019). Nevertheless, in a recent study only a research-grade gel-based system (e.g., compared to dry EEG) was able to simultaneously capture several expected EEG-based patterns (Radüntz, 2018; see Fig. 3), underscoring the importance of multiple metrics in assessing a system’s utility. Moreover, dry electrodes in their current form appear to be particularly sensitive to motion artefacts and consistently outperformed by gel-based systems (Oliveira et al., 2016; Radüntz, 2018; Zerafa et al., 2018). This may be because the use of electrodes through hair is more susceptible to signal degradation and movement artefacts (Chi et al., 2012), hence dry EEG requires further work (Chi et al., 2010; Ratti et al., 2017).

**Fig. 4:**
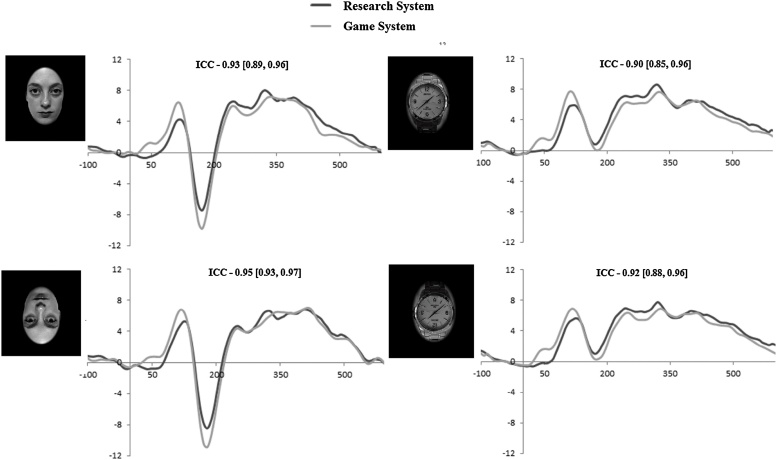
Example of face-sensitive N170 ERP assessed using a consumer-oriented mobile and wireless EEG system (*EPOC* by *Emotiv*; see also Fig. 1 and Table 2). Compared to a research-grade wired EEG system, this mobile system showed excellent interclass correlations in recording N170 amplitude/latency. The N170 recorded in both system showed higher amplitude in response to faces versus non-faces (e.g., watches), and longer latencies in response to upright faces versus inverted faces. Data presented here were restricted to channel P8 for simplicity (though data in channel P7 showed a similar pattern) and because it was where the grand-averaged peak amplitude was highest. Adapted from de Lissa et al. (2015).

Another type of study directly compared the data produced by mobile versus traditional systems (providing support for criterion validity) using indices such as intraclass correlations (ICC) to evaluate their degree of similarity, with >.75 indicating excellent agreement (Cicchetti, 1994). In the time domain, a consumer-oriented system (*EPOC*; see Table 1) was able to produce equivalent patterns on face-sensitive N170 ERP as with a traditional system (ICC = .89–95) (de Lissa et al., 2015; see Fig. 4). Similar promising results have been found for averaged ERP waveforms such as N2 and P3 (ICC = .74–92) in oddball tasks in adults (Badcock et al., 2013; Barham et al., 2017) and children (Badcock et al., 2015). However, some mobile systems may capture other features, such as mismatched negativity, with lower agreement (ICC = .44–74); require higher rejection rates (Badcock et al., 2015, 2013); and be outperformed in single-trial classifications – for example, of P3 (Duvinage et al., 2013) – by traditional systems.

#### 4.1.2 Reliability

Reliability broadly refers to the consistency of a measure across testing conditions. A recent study suggested that ‘session’ (of three different testing occasions) accounted for only little variance (1%) compared to ‘participants’ or ‘systems’ (Melnik et al., 2017), indicating that variability is driven by individual differences, although no metric was reported to assess each system’s reliability. Other studies have begun to incorporate indices of test-retest reliability. For example, good test-retest reliabilities have been reported for spectral features in auditory oddball tasks (more consistently with wet systems) during seated (ICC = .68–93) and walking conditions (ICC = .93–.99) (Oliveira et al., 2016), and similarly in eyes-open and eyes-closed resting states, for retesting one-day, one-week and one-month later (Ratti et al., 2017), and even with a single-electrode wireless system during eyes-closed states (ICCs = .76–.85). Nevertheless, reliabilities may be lower for eyes-open resting states or cognitive paradigms (ICCs = .57–.85) (Rogers et al., 2016), or for some dry systems due to misplacement of electrodes (Ratti et al., 2017).

For ERPs, good test-retest reliability for P3 amplitude can be obtained in an auditory oddball task with Pearson correlation r > .74 (Debener et al., 2015). Even while walking on a treadmill concurrent to a go/no-go task, good test-retest reliabilities were obtained for N2 amplitude/latency on average more than 2 years later (ICC > .64), although with lower reliabilities for P3 amplitude/latency (ICC = .32–.80) (Malcolm et al., 2017). Some mobile systems can be susceptible to delays/jitters due to the use of wireless transmission (Hairston et al., 2014), which can result in measurement error for time-based analyses, although some EEG features (e.g., Nc of negative central which consists of a slow wave) may not be necessarily affected by off-timing by a few msec. If needed, offline alignment corrections can be applied (Debener et al., 2015; Melnik et al., 2017; Ries et al., 2014). Nevertheless, several mobile (research-grade) systems now have hardware-based provision for integrating event triggers into the EEG data stream with minimal delay/jitter, potentially obviating the need for analytical compensations (Hairston et al., 2014; Ries et al., 2014).

**Fig. 5:**
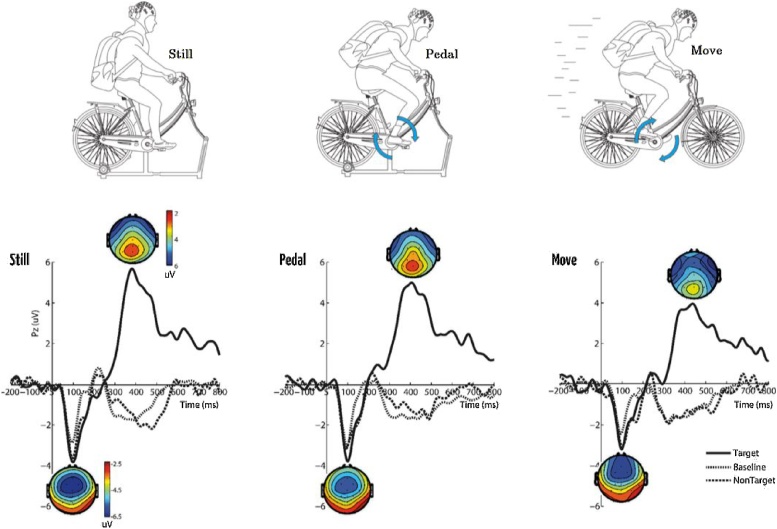
Grand average ERPs to three types of stimuli (Target, NonTarget and Baseline) for the three conditions of an auditory oddball task (including schematic for each condition; Still, Pedal and Move) at channel Pz, with topographies for N1 and P3 ERPs for the target stimuli (using *SMARTING* by *mBrainTrain*; also see Fig. 1 and Table 2). In the Still condition, participants performed one recording while sitting still on a bike in a fixed standard facing nature; in the Pedal condition, they performed one recording while pedalling on the bike while the bike remained in a fixed position; in the Move condition, they biked freely around on a 500 m course on a university campus. In all conditions, a clear posterior focus of the P3 topography is visible whereas the N1 is more central. P3 amplitude (but not N1 amplitude) in the Move condition was on average 31% and 26% lower as compared to the Still and Pedal conditions, respectively. Adapted from Zink et al. (2016).

A group of studies compared mobile systems during stationary versus non-stationary conditions using well-established paradigms, informing reliability across testing settings. Auditory oddball tasks (Debener et al., 2012; Scanlon et al., 2017; Zink et al., 2016) to elicit the P3 ERP can be performed in non-lab conditions. Even if significant artefacts may be present in a moving condition (i.e., biking freely in a university campus), above chance P3 single-trial classification is still possible (Zink et al., 2016; see Fig. 5). Similar promising data are emerging for use of mobile EEG during whole-body motion (e.g., Gramann et al., 2010), although not without additional analytical challenges (see Section 4.5).

#### 4.1.3 Overall assessment and suggestions for future steps

Validation studies for mobile EEG have used a multiplicity of methodologies. There is currently a lack of consensus on the most appropriate benchmarking criteria, making direct comparisons across studies/systems challenging, although some recent frameworks have been proposed (Melnik et al., 2017; Oliveira et al., 2016; Radüntz, 2018; Zerafa et al., 2018). It is clear that at least some current mobile systems can produce the expected signal, and that overall research-grade, gel-based systems (akin to traditional systems) offer the best signal quality, but potentially at a higher cost (see Sections 4.2 and 4.3 for other criteria for system selection). Nevertheless, some consumer-oriented systems can still be useful under certain circumstances (e.g., averaged ERPs). It remains to be shown whether more advanced techniques (e.g., connectivity and nonlinear approaches) can extract meaningful information from mobile EEG data beyond traditional analyses (Morán and Soriano, 2018). Moving forward, what would be most informative are systematic studies that report on both validity and reliability using multiple systems, paradigms and EEG parameters, and based on agreed benchmarking metrics as the field matures, to allow for systematic reviews/meta-analyses. Neurodevelopmentalists may also wish to test these systems independently if the information needed is unavailable, or to clarify potential developmentally-sensitive adaptations required for task/protocols, for example, the necessary number of trials to obtain a stable EEG signal in infants (Webb et al., 2015).

Opportunities could arise from dialogue between neurodevelopmentalists and manufacturers to share insights regarding the ideal mobile system given a specific population/purpose. This dialogue could draw from recent successful efforts such as the Lab Streaming Layer technology (Delorme et al., 2011), an academia-initiated standard to synchronize timing across platforms (e.g., EEG, eye-tracking, motion capture, etc.,) in which now many manufacturers participate, dramatically facilitating mobile EEG research (Kothe and Makeig, 2013; Ojeda et al., 2014). Our field can also take inspiration from existing open hackathons (e.g., bringing engineers and neurodevelopmentalists to collaborate intensively to produce a final hardware/software) and other related workshops tailored to neurodevelopmental questions.

### 4.2 Selection of electrode density

Low-density systems may be more affordable, comfortable and lightweight, but not necessarily suitable for all types of research. Some systems do not have sensors on the midline (Maskeliunas et al., 2016) or occipital sites (Radüntz, 2018), while others are placed around the ear (Bleichner and Debener, 2017; Debener et al., 2012; Mirkovic et al., 2016).

Reduced scalp coverage might constraint what processes can be studied and what analyses can be performed, although there is emerging evidence that even ear-based coverage can capture brain-based EEG features (Bleichner and Debener, 2017). Sufficient scalp coverage is critical for source localisation (Akalin et al., 2013). It can also be important for signal quality (as discussed in Section 4.1), reliable reference/re-reference scheme, and effective artefact rejection analyses (Picton et al., 2000), including muscle artefacts during motion-based paradigms (Reis et al., 2014; also see Section 4.5).

Higher-density systems may allow for more flexible applications across paradigms/analyses, and higher sensitivity to capture multiple brain sources of varying positions/orientations. Lower-density systems could sometimes be more advantageous, such as quicker set-ups with minimal preparation in a large sample of neonates. It remains to be seen whether the longevity of and the maintenance required for current research-grade mobile systems necessarily outweigh investment in a traditional EEG lab. However, the associated costs are likely to decrease over time.

### 4.3 Comfort issues

The ability to tailor to the various cognitive, sensory and motor characteristics of neurodevelopmental populations varies greatly across available systems. It is important to flexibly adjust to different head sizes/shapes without compromising set-up quality, as for example extreme outliers in head size can be common in some neurodevelopmental populations (Miles et al., 2005). Some consumer-oriented systems – primarily developed for everyday use – can produce position errors due to design issues, such as electrode size, fixed lengths and rigid structures (Hairston et al., 2014).

Further, dry EEG can be uncomfortable (sometimes causing skin irritation). Because dry electrodes exert pressure to ensure direct contact with the scalp, these can become tangled with long/thick hair (Hairston et al., 2014). Dry systems – at least in their current form – are thus not appealing for extensive periods of data collection. Non-contact EEG is promising but not available yet for routine use.

Recent studies evaluating mobile EEG have increasingly considered the user experience (Hairston et al., 2014; Melnik et al., 2017; Zerafa et al., 2018). This approach would also be important when evaluating a system’s suitability for neurodevelopmental populations, to inform practical issues such as whether it would indeed improve recruitment rates (e.g., is the system more appealing?), reduce attrition rates (e.g., is it tolerated for longer?) and minimise the need for desensitization protocols (e.g., does it look less ‘scary’?).

### 4.4 Considerations for large-scale data collection

The use of consumer-oriented low-density mobile EEG systems for efficient data collection is promising (Kontson et al., 2015), but it remains to be seen if the same applies to the use of research-grade high-density systems in neurodevelopmental populations while maintaining high signal quality. Not every system would suit this purpose. The choice of system would depend on headset-specific characteristics (e.g., shelf-life and battery duration) and also the weight of the broader ‘mobile’ kit (e.g., devices for stimuli presentation, EEG monitoring and data storage; cameras; and in-person interview materials). Smartphone-based EEG could be helpful to minimise equipment burden (Piñeyro Salvidegoitia et al., 2019; Stopczynski et al., 2014a, 2014b). For some types of EEG studies, a ‘mobile lab’ might be the most viable solution (e.g., studies involving large-scale nation-wide data collection in twin siblings). Concrete evidence for increased efficiency and cost-effectiveness compared to past studies using similar designs with traditional EEG would be informative.

**Fig. 6:**
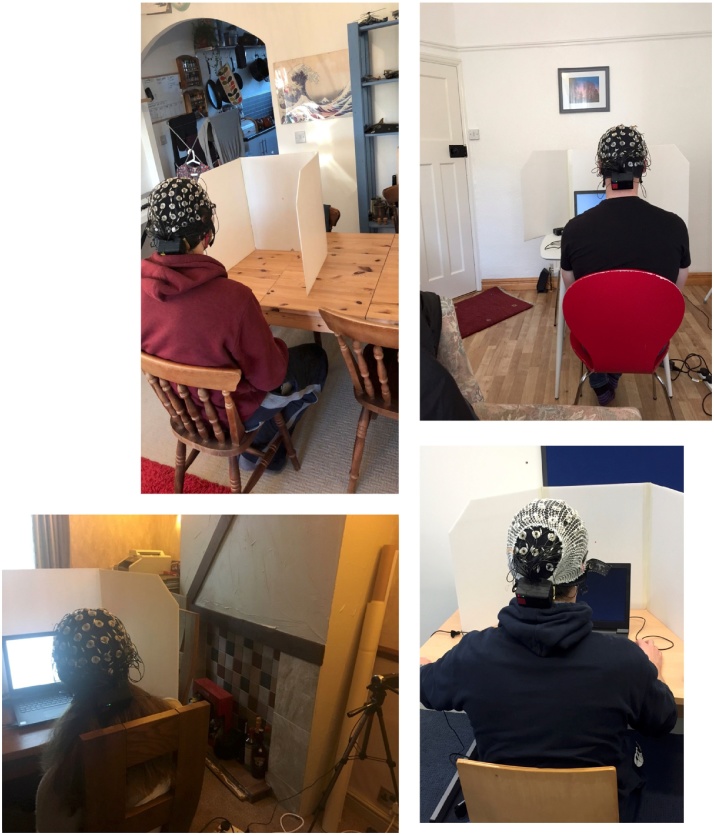
Illustration of large-scale mobile EEG testing across indoor locations using conventional stimuli presentation. Photographs were taken as part of regular research documentation to fine-tune testing protocol and inform standardization. Four mobile set-ups using a mobile EEG headset from *Cognionics* in clockwise order: testing in a home setting and of resting state recordings, which include folding panels to reduce visual distraction, having participants’ backs facing the window to reduce visual interference and having the researcher out-of-sight to allow for minimal interference and note-taking; testing also in a home setting with a similar set-up, this time of a task-based paradigm using stimuli delivery via a laptop; testing in an office setting, illustrating the use of a soft cotton-net to reduce cable movement if required; finally, testing in a home setting, with camera positioned to allow for recording of potential full-body movements/facial expressions for later revisions.

A key challenge is to devise protocols for testing in multiple/non-standardised settings, particularly when the impact of environmental variables on mobile EEG data remains unknown. There is little discussion about potential environmental noise (e.g., electromagnetic interference) generated from other devices that might be encountered in homes and schools (e.g., mobile phones, microwaves and air conditioning units). Note that the use of active electrodes (which can actively amplify voltage at the electrode), at least in traditional EEG systems, has shown to be useful in minimising such sources of noise (Kappenman and Luck, 2010; Metting van Rijn et al., 1990) and are increasingly incorporated into mobile EEG systems. Further, active shielding in many mobile systems appears to protect from 50/60 Hz ‘line’ noise interference (Bateson et al., 2017). While such electromagnetic interference may be unsystematic and has little impact overall, it remains an important issue to be considered more thoroughly as EEG testing for routine scientific research becomes increasingly performed in several set-ups within the same study. Further to the already excellent guidelines for EEG research (Picton et al., 2000; Webb et al., 2015) which contain many relevant points for mobile EEG studies, we propose some additional suggestions (Table 3) drawing from some initial experience with a mobile EEG system in a large-scale neurodevelopmental study (Fig. 6).

**Table 3:** Some Good Practice Suggestions for Mobile EEG Testing in Large-scale Studies with Several Non-Standard Settings.

Before the visit	• Decide on the minimum requirements for testing, e.g.,: - availability of a quiet space - availability of desk/table of appropriate size • Establish the additional features that would enhance standardisation, e.g.,: - space-layout that allows the researcher to be completely out of sight of the participant - available space to position a video camera to monitor the participant’s whole body and facial expressions during testing • Conduct a pre-visit interview (e.g., by phone/email/text) to: - ascertain environmental conditions to pre-empt challenges (e.g., other people who may be at home that day; sources of ambient noise; large windows without curtains which may allow for distractions; sources of lighting; location of plug sockets, etc.) - consider sending a video of the mobile system (and preparation process) prior to the visit to elicit potential concerns or special considerations
During the visit	• Maximise signal quality by using additional devices from the ‘mobile lab kit’ (note that these suggestions would not be suitable for every study/population, and some may be stress-inducing for certain participants): - inner headphones or foldable panels to reduce visual/auditory distractions - portable mini-fans to regulate room temperature - ventilation vests to regulate body temperature (during motion) - double-layered caps (or alternatively using soft cotton bandages) to restrain cables between layers and minimise cable movement • Take note of any deviations from standard protocol and annotations of events given the typical higher rates of modifications needed to work with neurodevelopmental cohorts • Document the general set-up in images (e.g., with photographs or videos; also see Fig. 6) • Perform active and continuous monitoring of the participant and of the EEG recording (both on-task and off-task) to ensure compliance levels
After the visit	• Review notes and visual documentations as a team to: - continue with protocol refinement and standardization - reinforce a culture of joint problem-solving and good scientific practice (critical for early use of this technology) - identify retrospectively unexpected noise sources (e.g., including potential interference from electronic devices) - facilitate characterisation of the environment into quantifiable variables to be considered in later analyses. • Store and archive EEG data in line with community-agreed standards to facilitate open science

*Note*. This list is not meant to be final or exhaustive and is only based on one group’s initial experience with these new technologies.

Given that we lack consensus guidelines, creative (but sensible) solutions are paramount, ideally with input from developmental/clinical experts. As mobile EEG becomes more widespread in neurodevelopmental research, it would be an asset to refine such protocols/guidelines by sharing advice across research groups, to help ensure protocol fidelity in multi-site collaborations and to encourage cross-lab replications. The latter would greatly benefit from developing EEG data sharing standards, such as following similar initiatives on developing brain imaging data structure (BIDS) for magnetic resonance imaging (MRI; Gorgolewski et al., 2016) and magnetoencephalography (MEG; Niso et al., 2018) to facilitate open data-sharing.

### 4.5 Considerations for motion-based paradigms

Participants’ motion inevitably introduces novel types of artefacts (for a more detailed review see Reis et al., 2014). Emerging evidence indicates that movement in motion-based mobile EEG paradigms can be dealt with given the appropriate technical and analytical approaches (Gramann et al., 2010; Gwin et al., 2010; Malcolm et al., 2015; Nathan and Contreras-Vidal, 2015; Wagner et al., 2014), for example drawing from advances in simultaneous fMRI-EEG data analyses which combine channel-based and source-based approaches (Gwin et al., 2010). Further evidence is needed for the applicability of these methods to neurodevelopmental research, especially if systems with lower sampling-rates are used (<128–250 Hz), which could constraint feature extraction (Reis et al., 2014). The advent of routine multi-modal data acquisition – including the incorporation of motion sensors using inertia measurement unit (IMU) acquisition alongside mobile EEG – would further contribute to the improvement of artefact-correction procedures (Reis et al., 2014).

Several challenges remain for designing non-stationary paradigms for neurodevelopmental research. One approach has been to attempt to model real-world complexities in the laboratory (Fig. 2). Such set-ups tend to be restricted to dedicated laboratories involving cumbersome/heavyweight equipment and no fully head-mounted headsets, including relatively large amplifiers stored in rucksacks worn by participants or attached to their waists (Banaei et al., 2017; Jungnickel and Gramann, 2016). Such paradigms can be impractical and uncomfortable for neurodevelopmental populations. Many of these also constitute proof-of-concept studies and are concerned primarily with the impact of different types of motions (Banaei et al., 2017; Doppelmayr et al., 2012; Jungnickel and Gramann, 2016; Zink et al., 2016). Thus, translations are pending for mobile paradigms that are appropriate and specific for interrogating developmentally-relevant neurocognition.

An alternative approach has been to introduce experimental manipulations within everyday settings, which faces the challenge of how to maximise control of stimuli delivery (Fig. 2). Creative solutions have included the presentation of auditory stimuli on headphones while cycling around a limited area (Zink et al., 2016), and of to-be-memorised word stimuli while participants walked on a pre-established route shown using a tablet screen held by the researcher (Griffiths et al., 2016). Smartphone-based stimuli presentation is also increasingly being incorporated into outdoor-based studies (Piñeyro Salvidegoitia et al., 2019). A smartphone-based mobile EEG system was worn for up to seven hours (Debener et al., 2015), further emphasising the possibility of real-world long-lasting recording. Nevertheless, these examples still consider ‘simple’ stimuli. Further mobile studies can incorporate more naturalistic/complex stimuli such as moving faces (Leong et al., 2017) or using turn-taking paradigms (Liao et al., 2015).

A particularly exciting possibility is the advent of truly naturalistic paradigms to link EEG activity with spontaneous behaviours. These could leverage existing methods for comprehensive moment-to-moment coding and annotation (Bakeman and Gottman, 1997; de Barbaro et al., 2013), together with advances in automated event detection/labelling using additional everyday sensors (Mohr et al., 2017) and even from the EEG signal itself (Su et al., 2018). These numerous possibilities may signify a paradigm shift away from reliance on lab-based protocols, but not without substantial challenges for methods (e.g., potential channel movement if the cap shifts in a free-moving interaction), as well as for analyses and interpretations. The initial adaptation/validation of well-established stationary cognitive paradigms using emerging EEG systems in everyday settings (e.g., and in large samples) may serve as a springboard to refine theories, hypotheses, and methodologies. This approach could then guide the development of mobile EEG paradigms in everyday settings with free-moving conditions in response to real-world stimuli (e.g., to study social communication in a real-life interaction or executive functioning in a shopping task).

## 5 Conclusions and final reflections

EEG technologies marked the historical beginning of human neuroscience research to study brain activity, and now stand firmly as one of the most accessible and flexible tools to study the brain in real-time given recent advances in mobile applications. Together with progress in signal processing, mobile EEG systems hold promise to advance developmental psychopathology research, particularly by increasing the overall ease of use of EEG technologies for neurodevelopmental populations, facilitating routine large-scale neural data-collection in powerful developmentally-informative studies, and inspiring the development of novel paradigms for studying neurocognition beyond typical stationary laboratory-based tasks. By maximally exploiting these opportunities, the field may draw closer to unravelling the aetiology and mechanisms of psychopathology across the lifespan.

Despite the recent proliferation of studies leveraging mobile EEG, the application of these advances in neurodevelopmental disorders is still in its infancy. Many mobile EEG systems are available currently, each with its strengths and drawbacks. We identified key challenges that remain for mobile EEG technologies to fully integrate both the relevant hardware and software advances into neurodevelopmental research. Signal quality is promising (in at least certain mobile systems), but we need more systematic studies and additional validations in neurodevelopmental populations. Considerations should also be given to electrode density and headsets’ comfort, as these aspects could constrain signal quality. Integration of mobile EEG into large-scale data collection in everyday settings is now feasible, and shared guidelines for standardisations across neurodevelopmentalists are encouraged. Finally, the advent of paradigms that examine real-world behaviours in everyday settings is an exciting possibility, but may require careful design to ensure good signal quality and neurodevelopmental relevance.

Increased input from neurodevelopmentalists would be an asset to further tailor mobile EEG advances for neurodevelopmental research and provide critical information for colleagues in the field. How current forms of mobile EEG can be used would depend on many factors, hence the decisions on EEG system selection would ultimately lie in the individual research group. For example, there is no reason why a neurodevelopmentalist could not start validating the use of some new EEG systems in a small sample of participants with specific neurodevelopmental disorders and using a well-established cognitive paradigm to study a novel EEG parameter for which information is lacking (e.g., time-frequency or network analyses). Alternatively, for neurodevelopmentalists who have access to signal-processing colleagues and collaborative links with manufacturers, it would be exciting to embark on a relatively ambitious and larger-scale project with mobile EEG, as they would in principle have the necessary expertise to address uncharted challenges. Continued innovation is critical, and researchers can begin to explore these technologies while being mindful of caveats.

Several challenges lie ahead for mobile EEG advances to be maximally exploited for neurodevelopmental research. Advances in hardware and software are clearly rapidly evolving. With increased input from neurodevelopmentalists in mobile EEG developments, mobile EEG technologies could be at the forefront of neurodevelopmental and psychopathology research in the years to come.

## Declarations of interest

None.

## Funding

The research was funded by the United Kingdom Medical Research Council (MRC) [Grant number: MR/N013182/1].

## Authors’ contributions

ALZ wrote the draft. ML and GM contributed to revisions.
